# Thigh fat and muscle each contribute to excess cardiometabolic risk in South Asians, independent of visceral adipose tissue

**DOI:** 10.1002/oby.20796

**Published:** 2014-05-26

**Authors:** Sophie V Eastwood, Therese Tillin, Andrew Wright, Jamil Mayet, Ian Godsland, Nita G Forouhi, Peter Whincup, Alun D Hughes, Nishi Chaturvedi

**Affiliations:** 1 National Heart and Lung Institute, Imperial College LondonLondon, UK; 2 Department of Medicine, Imperial College Healthcare NHS TrustLondon, UK; 3 Department of Endocrinology and Metabolic Medicine, Imperial College LondonUK; 4 MRC Epidemiology Unit, University of CambridgeCambridge, UK; 5 Division of Population Health Sciences and Education, St. George's University of LondonLondon, UK

## Abstract

**Objective:**

To compare fat distribution and associations between fat depots and cardiometabolic traits in South Asians and Europeans.

**Methods:**

Five hundred and fourteen South Asians and 669 Europeans, aged 56-86. Questionnaires, record review, blood testing, and coronary artery calcification scores provided diabetes and clinical plus subclinical coronary heart disease (CHD) diagnoses. Abdominal visceral (VAT) and subcutaneous adipose tissue, thigh subcutaneous adipose tissue (TSAT), intermuscular and intramuscular thigh fat and thigh muscle were measured by CT.

**Results:**

Accounting for body size, South Asians had greater VAT and TSAT than Europeans, but less thigh muscle. Associations between depots and disease were stronger in South Asians than Europeans. In multivariable analyses in South Asians, VAT was positively associated with diabetes and CHD, while TSAT and thigh muscle were protective for diabetes, and thigh muscle for CHD. Differences in VAT and thigh muscle only partially explained the excess diabetes and CHD in South Asians versus Europeans. Insulin resistance did not account for the effects of TSAT or thigh muscle.

**Conclusions:**

Greater VAT and TSAT and lesser thigh muscle in South Asians contributed to ethnic differences in cardiometabolic disease. Effects of TSAT and thigh muscle were independent of insulin resistance.

## Introduction

Obesity is a key risk factor for burgeoning global levels of diabetes and cardiovascular disease, and central deposition of adipose tissue appears particularly harmful 1,2. Urbanized South Asian populations, resident in the Indian subcontinent and settled overseas, experience greater rates of cardiometabolic disease [diabetes, coronary heart disease (CHD) and stroke] than European populations 3,4, and ethnic differences in body composition may contribute to this 5,6.

Computer tomographic (CT) or magnetic resonance (MR) imaging can differentiate abdominal depots into visceral adipose tissue (VAT) and subcutaneous adipose tissue (SAT) compartments, and quantify depots outside the abdomen that may contribute to cardiometabolic risk. South Asians have greater VAT in some 7–10, but not all studies 11, less lower limb skeletal muscle 10, and less intramuscular adipose tissue than Europeans 9. In Europeans, thigh subcutaneous adipose tissue (TSAT) and thigh muscle have beneficial, and thigh inter- and intramuscular adipose tissue deleterious effects on insulin resistance 12–15, HbA_1c_ 16 and inflammation 17,18. There is a paucity of comparable data for South Asians. Previous studies comparing fat and muscle in South Asians and Europeans are limited by small numbers, exclusion of those with disease or medications, and recruitment by advertisement rather than population sampling 7–11. These factors may introduce bias by ethnicity, as disease and medication use differ markedly by ethnicity, making recruits unrepresentative of the population from which they came, and thus findings less generalizable.

We compared fat depots of the abdomen and thigh, as well as thigh muscle, in South Asians and Europeans, examined individual and combined associations with risk factors and cardiometabolic disease by ethnicity and determined whether body fat distribution accounted for ethnic differences in cardiometabolic disease.

## Methods

### Study sample

We used cross-sectional follow-up data from the Southall And Brent REvisited (SABRE) study, a population-based cohort of individuals from north-west London 19. All South Asians were first-generation migrants, 82% were born in the Indian subcontinent and 14% in East Africa. Participants aged 40-69 (*n* = 4857) were randomly selected from age- and gender-stratified general practitioner lists and workplaces at baseline (1988–1991), and were followed-up between 2008 and 2011, aged 56-85 years (*n* = 4196). This analysis is restricted to 669 European and 514 South Asian participants who attended follow-up clinic (2008–2011). All participants gave written informed consent. Study approval was obtained from St Mary's Hospital Research Ethics Committee (07/H0712/109).

### Clinic measurements

Fasting blood samples were taken. Participants without diabetes underwent an oral glucose tolerance test. HOMA2-IR 20 and the Matsuda index 21 were used as measures of insulin resistance. For details of lipid, glucose, insulin, HbA1_c_, CRP, and IL-6 assays, see supplementary file.

Physical activity comprised the total weekly energy expended (MJ) on sports, walking, cycling and daily activities, using questions based on the Allied Dunbar fitness survey 22 and energy expenditure estimates based on work by Durnin and Passmore 23,24.

At follow-up, measurements were undertaken by trained researchers at St Mary's Hospital London. Height was measured barefoot using a stadiometer, and weight wearing a hospital gown, using a Tanita TBF-410 MA body composition analyzer. This calculated fat %, fat mass (kg), and total weight (kg). BMI was calculated as weight (kg)/height(m)^2^. Waist circumference was measured halfway between the costal margin and the iliac crest. Hip circumference was measured at the greater trochanter. Thigh circumference was measured halfway between the hip crease and the patella.

Abdominal and mid-thigh fat, and thigh muscle, were measured by computer tomography (CT) scan at 125kV with a Philips MX 8000 IDT64 detector. Images were taken with the participant supine with extended arms. VAT, and abdominal deep and superficial subcutaneous adipose tissue (DSAT, SSAT) were quantified from a single slice image of 10 mm thickness at the fourth lumbar vertebral level. We applied an attenuation range of −190 to −30 Hounsfield units (HU) to identify adipose tissue areas. VAT area was measured by circumscribing the visceral compartment manually [using Image-J software 25]. The abdominal fascial layer was demarcated, to enable calculation of DSAT and SSAT. A slice at mid-thigh level was used to quantify thigh fat and muscle areas. Muscle perimeter was delineated by an automated function. Thigh intermuscular adipose tissue (TIMAT) corresponded to the area in the attenuation range −190 to −30 HU, intramuscular or low attenuation muscle (LAM) to the area in the attenuation range 0-30 HU, and thigh muscle area of normal fat content (referred to as “thigh muscle” in analyses) to the area in the attenuation range 30-100 HU, as previously described 26. TSAT was then calculated by subtracting TIMAT from the total thigh area within the attenuation range −190 to −30 HU. Bone marrow fat was excluded. Right and left leg values were summed. Intra-observer reliability of VAT, SAT, TIMAT, and thigh muscle measurements was tested by comparing repeated measurements on 30 participants; coefficients of variation were 0.95%, 0.68%, 1.8%, and 2.7%, respectively.

Cardiac CT scanning was performed from the ascending aorta above the level of the coronary arteries to the inferior border of the heart. Transverse tomograms of 2.5 mm thickness were obtained with the participant in held inspiration. Coronary artery calcification (CAC) was quantified using proprietary software on a Philips Extended Brilliance computer workstation, and calcification was defined as an area >1 mm^2^ of density >130 HU. The CAC score was calculated as the sum of all lesion scores [Agatston Units (AU)]. Scans were read by a single experienced observer blinded to participant ethnicity. Inter-observer reproducibility, comparing scores from a senior investigator (AW), and intra-observer reproducibility were assessed initially and at intervals during follow-up, using the same 20 CT scans. The intra-class correlation coefficient for intra and inter-observer measurements was 0.94.

### Identification of diabetes and CHD

Diabetes was identified from primary care record review (recorded diagnosis or medications for diabetes), participant questionnaire (recall of physician-diagnosed diabetes plus diagnosis year or diabetes medication) or, in those with undiagnosed diabetes, raised oral glucose tolerance test plasma glucose by WHO 1999 criteria 27.

CHD was defined from primary care record review adjudicated by two clinicians—diagnosis was based on symptoms, cardiac enzymes, electrocardiography findings, exercise test findings, and coronary revascularization procedures, as per ASCOT (Anglo-Scandinavian Cardiac Outcomes Trial) criteria 28. Sub-clinical CHD was classified as a CAC score>400 AU 29 in those without diagnosed CHD. We used a composite measure of diagnosed CHD and subclinical CHD to address ethnic presentation/ diagnostic bias and to maximize power.

### Statistical analyses

Descriptive statistics were calculated for demographics, body composition, and cardiometabolic risk factors by gender and ethnicity. ANOVA and chi-squared tests determined ethnic group differences. Associations between body composition measures were assessed by Spearman's correlation coefficients.

Linear and logistic regression models examined associations between body composition measures and four cardiometabolic traits: diabetes, HbA_1c_, clinical and subclinical CHD (composite trait), and CRP. These were selected to represent prevalent disease (diabetes, CHD), and one continuous variable (HbA_1c_ and CRP) associated with each disease known to be influenced by fat distribution. We examined age-adjusted univariate associations between body composition and cardiometabolic traits stratified by ethnicity. Interactions between ethnicity and associations of body composition measures with cardiometabolic traits were sought.

Multivariable analyses showed combined associations between body composition measures and cardiometabolic traits by ethnicity. Measures with the strongest effects (i.e. largest standardized beta in univariate analyses) were entered first, and others included sequentially and retained according to the size of effect and statistical significance. The final multivariable models contained only variables which were important associates of cardiometabolic traits for at least one ethnic group (*P* < 0.05); enabling cross-ethnic group comparison. Secondary models included HOMA2-IR (selected in preference to the Matsuda index as it best attenuated associations between body composition measures and cardiometabolic traits).

Finally we evaluated the ability of body composition variables to account for ethnic differences in diabetes and CHD. For these analyses, variables significantly associated with cardiometabolic traits in the previous ethnicity-stratified multivariable analysis were entered and retained according to their impact on accounting for the ethnic difference (represented by an odds ratio for the association of South Asian ethnicity with cardiometabolic traits). Significant (*P* < 0.05) ethnicity/body composition interactions from multivariable models were additionally entered into models. Final models were adjusted for HOMA2-IR.

To test for multicollinearity, we examined variance inflation factors (VIFs), using a threshold of VIF > 5 to indicate model instability 30. Sensitivity analyses were performed by additionally adjusting multivariable models for years of education, physical activity, resting heart rate, inflammatory markers (CRP and IL-6), and lipids (HDL, LDL and triglycerides) in turn. Additionally, as it has been suggested that ethnic differences in HbA_1c_ exist even after adjustment for metabolic risk factors 31, we repeated analyses using fasting glucose instead of HbA_1c_. Sub-group analyses stratified by diabetes status, gender, and age group (younger: 56-72 years, older: 73-86 years). Analyses were performed using Stata 12 (College Station, Texas).

## Results

South Asians had more diabetes, lower levels of physical activity, greater hyperglycaemia and insulinaemia, and a greater burden of clinical and subclinical CHD than Europeans (Table1).

**Table 1:** Demographics and cardiometabolic traits, by sex and ethnicity

	Men			Women		
	European	South Asian	*P*^a^	European	South Asian	*P*^a^
***n***	517	439	-	152	75	-
**Age, years**	70 ± 6	69 ± 6	0.01	69 ± 6	68 ± 6	0.19
**Ever smoked**, %	67	25	<0.001	51	5.0	<0.001
**Physical activity, MJ/week**	10.4 ± 4.7	9.6 ± 4.5	0.02	8.7 ± 3.8	7.4 ± 3.2	0.03
**Diabetes**, %	19	44	<0.0001	20	36	0.05
**Diabetes treatment**, %	11	33	<0.001	9	21	0.01
**HbA_1c_**, %	5.9(5.7-6.2)	6.3(5.9-7.0)	<0.0001	5.8(5.6-6.1)	6.1(5.9-6.8)	<0.0001
**HbA_1c_, mmol/mol**	41(39-44)	45(41-53)	<0.0001	40(38-43)	43(41-51)	<0.0001
**Fasting blood glucose, mmol/l**	5.1(4.8-5.6)	5.4(4.8-6.2)	<0.0001	4.9(4.7-5.4)	5.1(4.6-6.0)	0.62
**Fasting blood insulin, µIU/ ml**	9.0(5.7-13.7)	9.8(6.4-15.5)	0.08	7.7(5.0-11.0)	8.9(5.6-13.3)	0.29
**HOMA2-IR**^b^	1.15(0.70-1.70)	1.10(0.70-1.80)	0.83	0.90(0.60,1.30)	1.15(0.80,1.75)	0.01
**Matsuda index of insulin resistance**^b^	0.22(0.14-0.37)	0.30(0.20-0.53)	0.0001	0.22(0.13-0.31)	0.35(0.18,0.5)	0.0003
**Total cholesterol, mmol/l**	4.73 ± 1.10	4.39 ± 1.00	<0.0001	5.37 ± 1.10	4.93 ± 1.31	0.01
**HDL-cholesterol, mmol/l**	1.33 ± 0.3	1.27 ± 0.30	0.001	1.57 ± 0.38	1.48 ± 0.32	0.11
**Triglycerides, mmol/l**	1.18(0.86-1.58)	1.20(0.90-1.65)	0.14	1.18(0.87-1.58)	1.23(0.95,1.65)	0.31
**Lipid-lowering treatment**, %	52	69	<0.001	38	64	<0.001
**CAC score >400 AU**^c^, %	26	27	0.96	13	9	0.37
**Diagnosed CHD**, %	18	30	0.001	6	23	0.001
**Clinical or subclinical CHD**^d^, %	37	46	0.005	16	30	0.02
**IL-6, pg/ml**	1.9(1.6-2.1)	1.7(1.4-1.9)	0.30	1.7(1.3-2.0)	1.2(1.0-1.3)	0.78
**CRP, mmol/ l**	1.6(0.9-3.2)	1.3(0.6-5.3)	0.88	2.0(0.8-4.2)	2.3(1.1-4.6)	0.88

Data are mean ± SD or median(IQR) for continuous variables.

a *P* for ethnic difference.

b participants without diabetes, CAC=coronary artery calcification, AU=Agatston units.

c participants without diagnosed coronary heart disease (CHD).

d defined as CAC score >400AU in those without diagnosed CHD, or CHD.

South Asians were shorter, lighter with smaller body circumferences (Table2). However, larger waist:hip and waist:thigh ratios in South Asians, compared to Europeans, indicated a tendency towards greater central fat deposition in the former. Crude CT measures of VAT were greater in European than South Asian men, while DSAT, SSAT, and TSAT did not differ. But South Asians were shorter and had less total fat mass than Europeans, and when these factors were accounted for, South Asians had more VAT, DSAT, SSAT, and TSAT than Europeans. TIMAT and low attenuation (“fat-rich”) muscle (LAM) were lower in South Asians than Europeans; these differences persisted when the lesser amount of thigh muscle in South Asians was accounted for. Ethnic differences in thigh muscle area, TIMAT, or LAM persisted within tertile of physical activity (data not shown). However, when adjusted for height and fat mass, there were no ethnic differences in TIMAT and LAM. Correlations between body composition variables were similar in each ethnic group (Table S1, Supporting Information).

**Table 2:** Body composition measures, by sex and ethnicity

	Men			Women		
	European	South Asian	*P*^a^	European	South Asian	*P*^a^
**Height (cm)**	173 ± 7	167 ± 6	<0.0001	160 ± 6	153 ± 6	<0.0001
**Weight (kg)**	84 ± 15	74 ± 12	<0.0001	72 ± 16	66 ± 12	0.001
**BMI (kg/m**^2^	28 ± 4	26 ± 4	<0.0001	28 ± 5	28 ± 5	0.85
**Waist circumference (cm)**	101 ± 12	99 ± 10	0.0001	94 ± 13	96 ± 11	0.31
**Waist:hip**	0.99 ± 0.6	1.01 ± 0.06	<0.0001	0.90 ± 0.07	0.95 ± 0.09	0.0002
**Waist:thigh ratio**	1.97 ± 0.18	1.99 ± 0.19	0.09	1.74 ± 0.21	1.82 ± 0.24	0.009
**Total fat mass (kg)**	23 ± 9	19 ± 7	<0.0001	28 ± 11	25 ± 9	0.02
**Fat free mass (kg)**	60 ± 8	55 ± 6	<0.0001	44 ± 6	41 ± 7	0.002
**Total fat** %	27 ± 6	26 ± 6	0.001	38 ± 7	37 ± 7	0.43
**Abdominal visceral adipose tissue (cm^2^)**	245 ± 104	228 ± 89	0.01	156 ± 75	162 ± 65	0.50
***Adjusted for age, height, fat mass***	230 ± 104	244 ± 89	0.0003	158 ± 75	174 ± 65	0.04
**Abdominal deep subcutaneous adipose tissue (cm^2^)**	147 ± 62	151 ± 58	0.25	147 ± 62	159 ± 64	0.19
***Adjusted for age, height, fat mass***	136 ± 62	162 ± 58	<0.0001	151 ± 62	166 ± 64	0.05
**Abdominal superficial subcutaneous adipose tissue (cm^2^)**	76 ± 33	77 ± 32	0.47	144 ± 67	158 ± 66	0.13
***adjusted for age, height, fat mass***	71 ± 33	81 ± 32	<0.0001	149 ± 67	166 ± 66	0.0006
**Thigh subcutaneous adipose tissue (cm^2^)**	115 ± 53	114 ± 52	0.69	230 ± 103	253 ± 102	0.12
***adjusted for age, height, fat mass***	108 ± 53	121 ± 52	<0.0001	237 ± 103	262 ± 102	0.02
**Thigh intermuscular adipose tissue (cm^2^)**	5.5 ± 3.6	4.8 ± 2.8	0.0006	3.9 ± 2.7	3.9 ± 2.9	0.90
***adjusted for age, thigh muscle area***	5.7 ± 3.6	4.3 ± 2.8	<0.0001	4.0 ± 2.7	3.0 ± 2.9	0.02
***adjusted for age, height, fat mass***	5.1 ± 2.7	5.2 ± 2.7	0.48	4.0 ± 2.3	4.3 ± 2.5	0.58
**Thigh low attenuation muscle (cm^2^)**	46 ± 22	40 ± 18	0.0001	35 ± 17	35 ± 14	0.94
***adjusted for age, thigh muscle area***	47 ± 22	36 ± 18	<0.0001	35 ± 17	30 ± 14	0.03
***adjusted for age, height, fat mass***	43 ± 18	43 ± 18	0.74	35 ± 15	39 ± 16	0.15
**Thigh muscle (cm^2^)**	214 ± 41	185 ± 37	<0.0001	151 ± 26	118 ± 27	<0.0001
***adjusted for age, height***	212 ± 37	186 ± 37	<0.0001	149 ± 27	121 ± 29	<0.0001

Data are mean ± SD.

a *P* for ethnic difference, thigh measurements are right + left leg.

**Figure 1:**
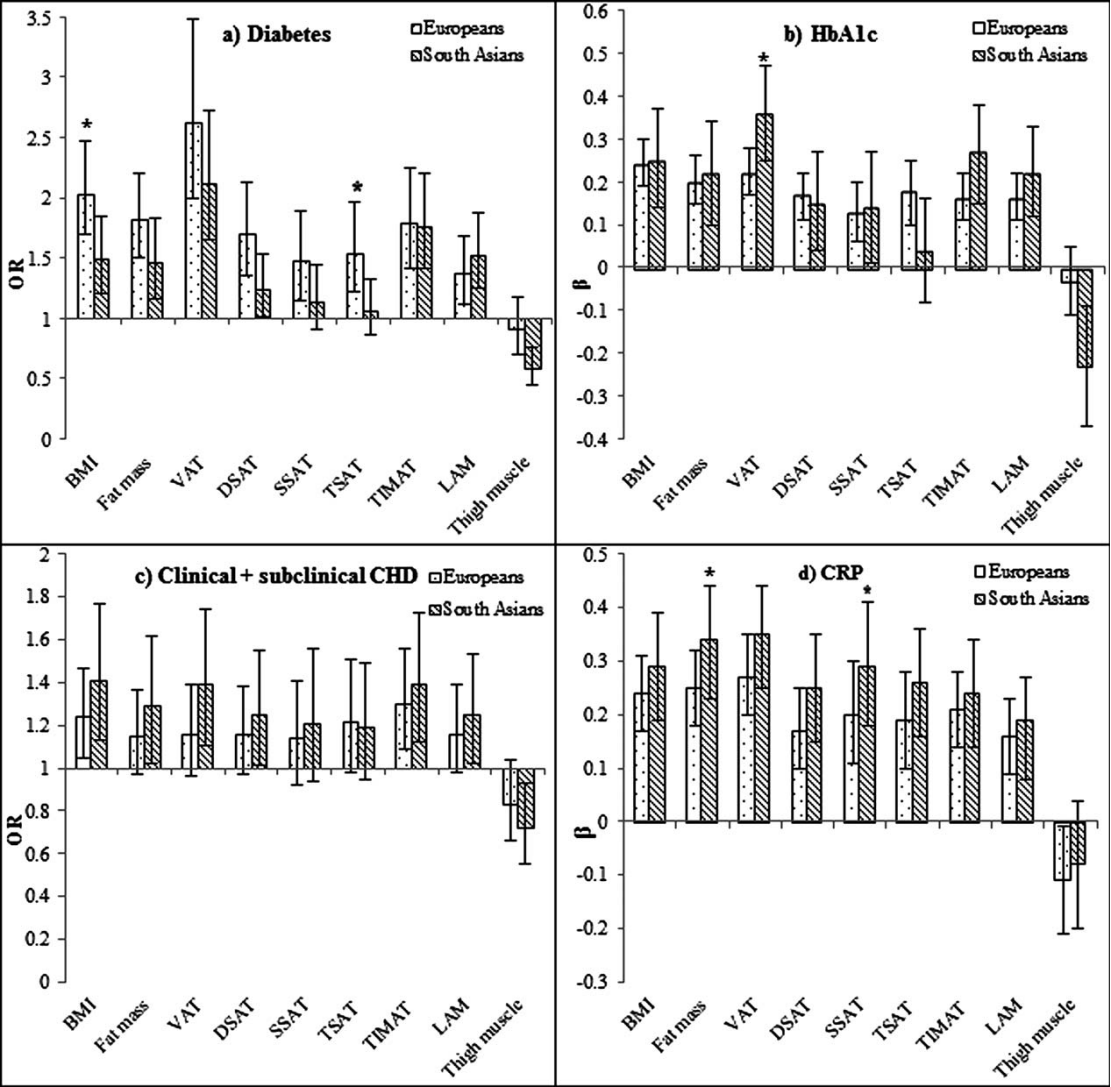
Associations between body composition measures and cardiometabolic risk by ethnicity: (a) Diabetes, (b) HbA_1c_, (c) Clinical + subclinical CHD, and (d) CRP.OR/standardized β for a 1SD increase of independent variables, adjusted for age and sex, 95% confidence intervals indicated by vertical lines, *ethnicity/ body composition measure interaction *P* < 0.05,VAT, abdominal visceral adipose tissue; DSAT, abdominal deep subcutaneous adipose tissue; SSAT, abdominal superficial subcutaneous adipose tissue; TSAT, thigh subcutaneous adipose tissue; TIMAT, thigh intermuscular adipose tissue; LAM, low attenuation muscle.HbA_1c_,CRP,VAT, DSAT, SSAT, TSAT, TIMAT, LAM log-transformed.

Taken separately, fat depots were positively associated with cardiometabolic risk factors (Figure 1), with VAT generally showing the strongest associations. Thigh muscle area was negatively associated with all cardiometabolic traits, significantly so in South Asians for diabetes, HbA_1c_, and clinical and subclinical CHD.

In multivariable models, VAT was the strongest independent correlate of diabetes, HbA_1c_, clinical and subclinical CHD and CRP, more so in South Asians than Europeans for HbA_1c_ (*P* = 0.01 for interaction) (Table3). After adjustment for VAT, TSAT became inversely associated with diabetes and HbA_1c_ in South Asians, and SSAT inversely associated with HbA_1c_ in Europeans, reversing positive univariate associations; all these were significant as interactions. Thigh muscle area largely retained inverse associations with diabetes, HbA_1c_, clinical and subclinical CHD and CRP, an effect greater for South Asians than Europeans for metabolic traits. Additional adjustment for HOMA2-IR (Table2), only attenuated VAT associations with clinical and subclinical CHD; associations remained significant for diabetes, HbA_1c_ and CRP. Furthermore, associations between other depots and all cardiometabolic traits remained as described above after adjustment for HOMA2-IR. TIMAT and LAM were not independently associated with cardiometabolic traits in multivariable models.

**Table 3:** Multivariable associations between body composition measures, insulin resistance and cardiometabolic traits, by ethnicity

	Europeans				South Asians			
Cardiometabolic traits	Model factors	β/ OR	95% CI	p	β/ OR	95% CI	p	p^a^
Diabetes	*VAT*	2.07	1.44,2.97	<0.001	2.10	1.53,2.89	<0.001	0.45
	*TSAT*	0.77	0.53,1.12	0.18	0.45	0.31,0.66	<0.001	0.002
	*Thigh muscle*	0.87	0.66,1.16	0.35	0.45	0.33,0.62	<0.001	0.04
Diabetes	*VAT*	1.80	1.23,2.63	0.002	1.58	1.13,2.23	0.008	0.85
	*TSAT*	0.74	0.50,1.08	0.12	0.46	0.31,0.68	<0.001	0.002
	*Thigh muscle*	0.85	0.65,1.13	0.27	0.43	0.31,0.59	<0.001	0.03
	*HOMA2-IR*	1.44	1.11,1.86	0.007	1.75	1.36,2.26	<0.001	0.26
HbA_1c_	*VAT*	0.17	0.09,0.26	<0.001	0.33	0.19,0.47	<0.001	0.002
	*SSAT*	−0.15	−0.27,0.03	0.01	0.07	−0.15,0.30	0.52	0.02
	*TSAT*	0.08	−0.05,0.21	0.21	−0.42	−0.64,−0.20	<0.001	<0.001
	*Thigh muscle*	−0.04	−0.12,0.04	0.31	−0.31	−0.45,−0.17	<0.001	0.003
HbA_1c_	*VAT*	0.11	0.02,0.20	0.02	0.16	0.02, 0.30	0.03	0.56
	*SSAT*	−0.11	−0.23,0.01	0.05	0.06	−0.16,0.28	0.58	0.08
	*TSAT*	0.05	−0.08,0.18	0.43	−0.38	−0.60,−0.17	<0.001	<0.001
	*Thigh muscle*	−0.04	−0.13,0.04	0.28	−0.30	−0.43,−0.16	<0.001	0.002
	*HOMA2-IR*	0.15	0.08,0.22	<0.001	0.35	0.24,0.47	<0.001	0.001
Clinical + subclinical CHD	*VAT*	1.08	0.84,1.41	0.54	1.32	1.00,1.75	0.05	0.27
	*Thigh muscle*	0.82	0.65,1.04	0.12	0.73	0.56, 0.94	0.02	0.37
Clinical + subclinical CHD	*VAT*	0.99	0.75, 1.28	0.88	1.10	0.80, 1.51	0.55	0.84
	*Thigh muscle*	0.82	0.64,1.04	0.10	0.70	0.53,0.93	0.01	0.37
	*HOMA2-IR*	1.23	1.00,1.54	0.06	1.45	1.14,1.84	0.002	0.27
CRP	*VAT*	0.17	0.07,0.27	0.001	0.25	0.13,0.37	<0.001	0.37
	*Thigh muscle*	−0.13	−0.22,0.03	0.009	−0.07	−0.20,0.04	0.21	0.60
CRP	*VAT*	0.17	0.06,0.28	0.002	0.25	0.11,0.38	<0.001	0.84
	*Thigh muscle*	−0.12	−0.23,0.02	0.01	−0.07	−0.19,0.05	0.25	0.58
	*HOMA2-IR*	−0.01	−0.11,0.07	0.73	0.01	−0.10,0.11	0.89	0.40

OR/standardised β for a 1SD increase of independent variables; age, sex, height, fat massadjusted.

a *P* for ethnicity interaction, VAT=abdominal visceral adipose tissue, SSAT=abdominal superficial subcutaneous adipose tissue, TSAT=thigh subcutaneous adipose tissue HbA_1c_, CRP,VAT, SSAT, TSAT log-transformed.

We then examined whether body composition could account for ethnic differences in disease (Table4). Ethnicity interaction terms were used for TSAT and thigh muscle in diabetes (but not CHD) models, in accordance with the above findings. VAT and separately thigh muscle attenuated the South Asian diabetes excess from an odds ratio of 3.78(2.80,5.10) to 3.56(2.62,4.83) and to 3.14(2.28,4.32), respectively, while TSAT increased it to 4.13(3.03,5.65). The effects of VAT were partly explained by insulin resistance, though those of TSAT and thigh muscle were not. For the South Asian excess of CHD [OR 1.76(1.33,2.33)], thigh muscle appeared to better account for the ethnic difference [OR 1.50(1.12,2.02) than VAT (OR 1.71(1.29,2.27)]. The effects of VAT, but not thigh muscle appeared attenuated when HOMA2-IR was entered into the model.

**Table 4:** Ethnic differences in diabetes and CHD, adjusted for body composition measures and insulin resistance

Model	Factors	Diabetes			Clinical plus subclinical CHD		
		OR	95% CI	*P*	OR	95% CI	*P*
**1**^a^	South Asian ethnicity	3.08	2.37,3.99	<0.001	1.63	1.28,2.08	<0.001
**2**^b^	South Asian ethnicity	3.78	2.80,5.10	<0.001	1.76	1.33,2.33	<0.001
**3**^b^	South Asian ethnicity	3.56	2.62,4.83	<0.001	1.71	1.29,2.27	<0.001
	VAT	2.09	1.65,2.63	<0.001	1.18	0.98,1.43	0.07
**4**^b^	South Asian ethnicity	3.19	2.33,4.37	<0.001	1.60	1.20,2.13	0.001
	VAT	1.72	1.34,2.20	<0.001	1.06	0.87,1.30	0.55
	HOMA2-IR	1.59	1.32,1.90	<0.001	1.35	1.14,1.59	<0.001
**5**^b^	South Asian ethnicity	3.14	2.28,4.32	<0.001	1.50	1.12,2.02	0.007
	Thigh muscle	0.78	0.61,0.98	0.04	0.78	0.65,0.93	0.006
	Thigh muscle#ethnicity	0.91	0.69,1.21	0.53	-	-	-
**6**^b^	South Asian ethnicity	2.65	1.90,3.69	<0.001	1.35	1.00,1.84	0.05
	Thigh muscle	0.74	0.58,0.94	0.01	0.76	0.63,0.91	0.003
	Thigh muscle#ethnicity	0.90	0.67,1.21	0.50	-	-	-
	HOMA2-IR	1.82	1.52,2.17	<0.001	1.38	1.18,1.62	<0.001
**7**^b^	South Asian ethnicity	4.13	3.03,5.65	<0.001	-	-	-
	TSAT	0.77	0.57,1.03	0.08	-	-	-
	TSAT#ethnicity	0.76	0.58,0.99	0.05	-	-	-
**8**^b^	South Asian ethnicity	3.60	2.61,4.97	<0.001	-	-	-
	TSAT	0.79	0.58,1.07	0.13	-	-	-
	TSAT#ethnicity	0.73	0.55,0.97	0.03	-	-	-
	HOMA2-IR	1.78	1.49,2.12	<0.001	-	-	-
**9**^b^	South Asian ethnicity	2.60	1.86,3.63	<0.001	1.34	0.99,1.83	0.06
	VAT	1.72	1.33,2.21	<0.001	1.07	0.87,1.31	0.55
	Thigh muscle	0.74	0.59,0.94	0.02	0.76	0.63,0.91	0.003
	Thigh muscle#ethnicity	0.91	0.68,1.23	0.55	-	-	-
	HOMA2-IR	1.61	1.34,1.94	<0.001	1.36	1.15,1.61	<0.001

OR for a 1SD increase of independent variables.

a age, sex-adjusted.

b age, sex, height, fat mass-adjusted.

VAT=abdominal visceral adipose tissue, TSAT=thigh subcutaneous adipose tissue, VAT, TSAT, HOMA2-IR log-transformed.

There was no indication of multicollinearity in multivariable models. Results were similar when multivariable models were adjusted for years of education, physical activity, resting heart rate, inflammatory markers, or lipids. Additionally, replacing fasting glucose with HbA_1c_ produced identical results. Sub-group analyses demonstrated persistence of patterns, regardless of diabetes status, gender, or younger/ older age group.

## Discussion

South Asians had more visceral and subcutaneous abdominal and thigh fat than Europeans, even when their smaller frame size was accounted for. VAT had the greatest adverse impact on cardiometabolic risk factors and disease, while TSAT and thigh muscle were independently protective. Adverse effects of VAT on HbA_1c_, and protective effects of TSAT and thigh muscle on diabetes and HbA_1c_ were stronger in South Asians than in Europeans. Ethnic differences in VAT, TSAT, and thigh muscle contributed to, but only partly accounted for, ethnic differences in diabetes and CHD. Effects of TSAT and thigh muscle were independent of insulin resistance.

We accounted for ethnic differences in body frame size and depot-specific lean mass when comparing ethnic differences in fat depots. It was only after allowing for their shorter height and lesser total fat mass that we found South Asians had relatively more VAT, DSAT, SSAT, and TSAT than Europeans. This is a standard approach when using surface anthropometry, for example by indexing waist circumference to hip circumference or height, and is recognized by some previous studies of ethnic differences in abdominal fat using imaging, comparing African American and white American populations 32. Comparisons between South Asians and Europeans have generally not employed this method, but some matched on BMI or weight and thus may have allowed for body size differences 8,10.

Our findings of greater VAT in South Asians than Europeans are consistent with most 7–10, but not all studies 11. We are not aware of other studies comparing CT-measured TSAT in South Asians and Europeans, though larger thigh skinfolds in South Asians have been previously reported 33. We expected South Asians to have higher intermuscular (TIMAT) and intramuscular (LAM) fat than Europeans, as these depots are thought to have adverse effects on metabolic risk 12,13. However, South Asians had less of both the depots, or similar amounts when differences in height and fat mass were accounted for, when compared with Europeans. Consequently, we considered the possibility that Europeans may have had more inter/intramuscular fat than South Asians because of their higher levels of physical exercise [these ethnic differences in exercise have been previously noted 34]. A paradoxically raised intramyocellular lipid content (IMCL) is seen in endurance-trained athletes 35. Yet both ethnic groups showed inverse relationships between each fat depot and physical activity. South Asians had less thigh muscle lean tissue when compared with Europeans, reflecting previous findings 10.

Most ectopic depots had adverse effects on cardiometabolic risk. VAT was the strongest independent correlate of cardiometabolic risk and was more strongly associated with HbA_1c_ in South Asians than Europeans. This fits with the results of a small study (*n*=20) which indicated that VAT may have a more marked adverse effect on insulin resistance in South Asians than Europeans 10. Previous work comparing associations between VAT and CRP in Europeans and South Asians showed similar effects in both groups 36, though our findings suggest the associations may be stronger in South Asians. South Asians have larger, less insulin-sensitive adipocytes than Europeans 9,11. Thus adverse functional capacity coupled with greater amounts of VAT in South Asians may contribute in tandem to their excess cardiometabolic risk 9.

We and others have shown that TSAT is protective for diabetes and HbA_1c_, but this association only emerges when VAT is accounted for 12,16. In addition, we demonstrated that protective effects of TSAT are greater in South Asians than Europeans, though it is unclear why. As far as we are aware, no studies have compared thigh adipocytes in these groups, and favorable differences in South Asians may exist, as unfavorable ones do for abdominal adipocytes 9,11. Matching our findings, previous studies have reported detrimental associations between TIMAT or LAM and metabolic risk factors, which do not remain after adjustment for abdominal fat depots 12,15,17.

Thigh muscle appeared to be protectively and independently associated with diabetes, HbA_1c_, and CHD in South Asians, and CRP in Europeans. Muscle mass has previously been shown to be negatively associated with inflammatory markers 18. Previous studies have shown that thigh muscle density (in Europeans) and total lean muscle mass (in South Asians) are positively associated with insulin sensitivity 14,37; suggesting this as a mechanism for muscle being protective for cardiometabolic traits. However, when we adjusted for insulin resistance, thigh muscle retained beneficial associations with HbA_1c_ and CHD—though there may have been residual confounding as calculated measures of insulin resistance are imprecise. Another explanation for these cross-sectional findings is reverse causality, i.e. diabetes/CHD leading to reduced physical activity, and thus reduced muscle area. However, in sensitivity analyses where multivariable models were adjusted for physical activity, protective associations for thigh muscle remained. Additionally, strong negative linear associations between thigh muscle and HbA_1c_ were observed, well into the normoglycaemic range, arguing against reverse causality. As there is evidence that lower cardiorespiratory fitness in South Asians when compared to Europeans may explain their adverse metabolic profile 33, we further adjusted multivariable models for heart rate, as a proxy for fitness. This did not alter the results, though the lack of a direct measure of cardiorespiratory fitness is a limitation of this study. The greater beneficial effects of muscle on metabolic measures in South Asians compared with Europeans are unexplained.

Our earlier longitudinal analysis showed that central obesity, as measured by waist-hip ratio, only partly accounted for the excess diabetes incidence in South Asians 5. We explored this association using the more precise measure of VAT, and again demonstrated some attenuation of the ethnic difference. Thigh muscle also explained some of ethnic difference when added to the model, as South Asians had less of this protective depot. Conversely, inclusion of TSAT increased the South Asian diabetes excess. This suggests that the greater amount of this apparently favorable depot provides some protection from diabetes in South Asians; bringing their levels of TSAT down to that of Europeans would increase their risk of diabetes. Adjustment for insulin resistance attenuated but did not abolish the relationship between VAT and diabetes, suggesting insulin resistance does not wholly mediate the association with diabetes. A novel finding is that the effects of TSAT and thigh muscle on the excess diabetes risk in South Asians were independent of VAT or insulin resistance. This suggests that the apparent protective effects of TSAT and thigh muscle act on a different pathway to the adverse effects of VAT, and warrants further exploration. It is possible that thigh muscle acts as a marker for cardiorespiratory fitness, which predicts outcomes independently of physical activity 38. Similarly, while VAT accounted for some of the ethnic difference in CHD, much of the difference remained unexplained, and the impact of VAT appeared to act via insulin resistance. The lesser thigh muscle in South Asians also contributed to their excess CHD, and, unlike VAT, its effect was independent of insulin resistance.

Strengths of this study include a comparatively large sample 8–12, accurate CT quantification of body composition measures, and the examination of many fat depots simultaneously on several cardiometabolic traits. We provide a more representative sample than previous similar research, as our participants were recruited at baseline (1988–1991) through population stratified random sampling, unlike other studies which limited their recruitment to healthy volunteers 7–11. These analyses were performed on participants at the follow-up clinic (2008–2011), thus bias may have been introduced by loss to follow-up, although proportions of baseline participants available for these analyses did not differ by ethnicity (30% of South Asians and 29% of Europeans). If we restricted our sample to those without previously diagnosed diabetes or CHD or not in receipt of lipid-lowering medication, only 26% of South Asians, and 49% of Europeans remained (ethnic difference *P* < 0.001). Such restrictions therefore have the potential to introduce important ethnic biases, and reduce generalizability of findings. Our population was relatively elderly, compared to previous studies 7–11, appropriate given this group suffer more cardiometabolic disease. Some associations may have altered as a result of disease or treatment. Measurement error may have been introduced by the use of bioimpedance to quantify body fat in South Asians—as far as we know, this method has not been validated in this group. This study only examined cross-sectional associations; therefore we cannot infer causality and further longitudinal research is needed to replicate the findings.

In summary, as well as greater levels of detrimental abdominal visceral adipose tissue (VAT), South Asians have greater levels of protective TSAT, and lesser levels of protective skeletal muscle than Europeans. In South Asians these depots act independently on cardiometabolic risk and their effects appear markedly greater than in Europeans. Insulin resistance contributed to the effects of VAT on cardiometabolic risk, but not to the effects of TSAT or thigh muscle. In combination, depots, particularly VAT and muscle, contributed to but could not wholly explain the South Asian excess of diabetes and CHD. Explanations for the marked protective effects of TSAT and muscle on cardiometabolic traits in South Asians should be urgently sought, as these may provide novel insights and targets for intervention in this high risk population.
