# Supplementary material for: Thigh fat and muscle each contribute to excess cardiometabolic risk in South Asians, independent of visceral adipose tissue
Source: Obesity (Silver Spring). 2014 May 26;22(9):2071–9. doi: 10.1002/oby.20796 (PMC4150020; doi:10.1002/oby.20796)
Supplement: Supplementary file 1 [file oby0022-2071-sd1.docx]

**Thigh fat and muscle each contribute to excess cardiometabolic risk in South Asians, independent of visceral adipose tissue**

**Authors:**

Sophie V Eastwood^1^, Therese Tillin^1^, Andrew Wright^2^, Jamil Mayet^1^, Ian Godsland^3^, Nita G Forouhi^4^, Peter Whincup^5^, Alun D Hughes^1^, Nishi Chaturvedi^1^

**Institutions:**

^1^National Heart and Lung Institute, Imperial College London, London, United Kingdom

^2^Department of Medicine, Imperial College Healthcare NHS Trust, London, United Kingdom

^3^Department of Endocrinology and Metabolic Medicine, Imperial College London, United Kingdom

^4^MRC Epidemiology Unit, University of Cambridge, Cambridge, United Kingdom

^5^Division of Population Health Sciences and Education, St. George’s University of London, London, United Kingdom

**Corresponding author:**

Dr Sophie V Eastwood, International Centre for Circulatory Health, National Heart and Lung Institute, Imperial College London, 59-61 North Wharf Road, London W2 1LA, United Kingdom. Tel: +44(0)20 75942955, fax: +44(0)20 75943392, email: [s.eastwood@imperial.ac.uk](mailto:s.eastwood@imperial.ac.uk)

**Supporting information**

**Details of biochemical assays**

Fasting plasma total cholesterol and triglycerides were analysed by peroxidase colorimetry and HDL-cholesterol using the accelerator-selective detergent method (both Abbott, Wiesbaden, Germany). Glycated haemoglobin (HbA_1c)_ was measured on whole blood using ion exchange liquid chromatography (Tosoh, San Francisco, USA). Fasting and 2-hour glucose were measured using a plasmahexokinase analyser (ARCHITECT c/AEROSET system, Abbott, Wiesbaden, Germany). Serum fasting and 2-hour insulin were measured by immunoassay (Elecsys2010, Roche, Burgess Hill, UK). C-reactive protein (CRP) was measured with an automated analyser (c311Roche, Burgess Hill, UK). Serum interleukin-6 (IL-6) was measured by electrochemiluminescent-immunoassay (MSD, Rockville, MD, USA).

**Table S1. Correlations between body composition measures, in a) Europeans and b) South Asians.**

Data are Spearman’s correlation coefficients, ^1^p<0.00^1^, ^2^p<0.01, ^3^ p<0.05. V^A^T=abdominal visceral adipose tissue, DSAT= abdominal deep subcutaneous adipose tissue, SSAT= abdominal superficial subcutaneous adipose tissue, TSAT= thigh subcutaneous adipose tissue, TIMAT=thigh intramuscular adipose tissue, LAM=low attenuation muscle.

|  | **BMI, kg/m²** | **Fat mass, kg** | **VAT, cm²** | **DSAT, cm²** | **SSAT, cm²** | **TSAT, cm²** | **TIMAT, cm²** | **LAM, cm²** | **Thigh muscle, cm²** |
| --- | --- | --- | --- | --- | --- | --- | --- | --- | --- |
| **BMI** | 1 |  |  |  |  |  |  |  |  |
| **Fat mass, kg** | 0.87^1^ | 1 |  |  |  |  |  |  |  |
| **VAT, cm²** | 0.66^1^ | 0.55^1^ | 1 |  |  |  |  |  |  |
| **DSAT, cm²** | 0.78^1^ | 0.77^1^ | 0.50^1^ | 1 |  |  |  |  |  |
| **SSAT, cm²** | 0.57^1^ | 0.69^1^ | 0.16^1^ | 0.64^1^ | 1 |  |  |  |  |
| **TSAT, cm²** | 0.59^1^ | 0.69^1^ | 0.12^2^ | 0.60^1^ | 0.82^1^ | 1 |  |  |  |
| **TIMAT, cm²** | 0.63^1^ | 0.54^1^ | 0.61^1^ | 0.51^1^ | 0.24^1^ | 0.27^1^ | 1 |  |  |
| **LAM, cm²** | 0.61^1^ | 0.52^1^ | 0.49^1^ | 0.48^1^ | 0.27^1^ | 0.32^1^ | 0.72^1^ | 1 |  |
| **Thigh muscle, cm²** | 0.13^2^ | -0.005 | 0.25^1^ | 0.08^3^ | -0.29^1^ | -0.38^1^ | -0.06 | -0.05 | 1 |

**b)**

|  | **BMI, kg/m²** | **Fat mass, kg** | **VAT, cm²** | **DSAT, cm²** | **SSAT, cm²** | **TSAT, cm²** | **TIMAT, cm²** | **LAM, cm²** | **Thigh muscle, cm²** |
| --- | --- | --- | --- | --- | --- | --- | --- | --- | --- |
| **BMI** | 1 |  |  |  |  |  |  |  |  |
| **Fat mass, kg** | 0.90^1^ | 1 |  |  |  |  |  |  |  |
| **VAT, cm²** | 0.55^1^ | 0.51^1^ | 1 |  |  |  |  |  |  |
| **DSAT, cm²** | 0.75^1^ | 0.75^1^ | 0.36^1^ | 1 |  |  |  |  |  |
| **SSAT, cm²** | 0.66^1^ | 0.69^1^ | 0.18^1^ | 0.62^1^ | 1 |  |  |  |  |
| **TSAT, cm²** | 0.69^1^ | 0.71^1^ | 0.17^1^ | 0.63^1^ | 0.84^1^ | 1 |  |  |  |
| **TIMAT, cm²** | 0.51^1^ | 0.49^1^ | 0.49^1^ | 0.46^1^ | 0.25^1^ | 0.28^1^ | 1 |  |  |
| **LAM, cm²** | 0.61^1^ | 0.54^1^ | 0.39^1^ | 0.52^1^ | 0.37^1^ | 0.44^1^ | 0.68^1^ | 1 |  |
| **Thigh muscle, cm²** | -0.01 | -0.06 | 0.11^3^ | -0.07 | 0.37^1^ | -0.42^1^ | -0.11^3^ | -0.19^1^ | 1 |
